# Retaining the long-survive capacity of Circulating Tumor Cells (CTCs) followed by xeno-transplantation: not only from metastatic cancer of the breast but also of prostate cancer patients

**DOI:** 10.18632/oncoscience.8

**Published:** 2013-12-31

**Authors:** Elisabetta Rossi, Massimo Rugge, Antonella Facchinetti, Marco Pizzi, Giorgia Nardo, Vito Barbieri, Mariangela Manicone, Stefania De Faveri, Maria Chiara Scaini, Umberto Basso, Alberto Amadori, Rita Zamarchi

**Affiliations:** ^1^ Department of Surgery, Oncology and Gastroenterology, Oncology Section, University of Padova, Padova, Italy; ^2^ IOV-IRCCS, Padova, Italy; ^3^ Department of Medical Diagnostic Sciences & Special Therapies, Surgical Pathology & Cytopathology Unit, University of Padova, Padova, Italy.

**Keywords:** Circulating Tumor Cells, EpCAM, prostate cancer, xenograft assay, breast cancer

## Abstract

We investigated whether Circulating Tumor Cells (CTCs) isolated from epithelial tumors could survive and grow in xenotransplants.

To this purpose, EpCAM-positive CTCs were enriched by CellSearch platform the only FDA-cleared automated platform that quantifies tumor burden in peripheral blood and provides clinical evidence of predictive and prognostic value. The CTCs were isolated from metastatic prostate (n=6) and breast (n=2) cancer patients. The xenograft assay was developed in 8-week-old NOD/SCID mice that were subcutaneously injected with increasing amounts of CTCs (ranging from 50 to 3000).

Human CTCs were found in 8 out of 8 murine peripheral blood (muPB) and in 6 out of 8 murine bone marrow (muBM) samples, after a median follow-up of 10.3 months. Six out of 8 spleens were positive for human cytokeratin. Our assay showed higher successful rate than those previously reported in breast cancer and hepatocellular carcinoma.

The role of EpCAM during carcinogenesis is controversial. The identification of human CTCs in muPB, muBM and spleen demonstrates that the EpCAM-positive fraction of CTCs retains the migratory capacity. This is the first experimental evidence that as few as 50 EpCAM-positive prostate cancer CTCs putatively contain metastasis-initiating-cells (MIC).

## INTRODUCTION

The presence of epithelial tumor cells in peripheral blood (PB) of cancer patients has been longtime associated with metastasis development [1, 2]. Recently, an inverse correlation between Circulating Tumor Cells (CTCs) burden and overall survival has been demonstrated in solid tumors [3-5]; moreover, changes in CTC counts have been associated with prognosis as early as the first treatment cycle [6-8].

Despite this clinical evidence, the tumorigenic potential of CTCs still remains to be proven. Several technical and conceptual hitches constrained a definitive demonstration of the CTC role in the metastatic process, such as the lack of an adequate niche to allow CTC growth, and a general consensus about the gold standard method to isolate these rare cells [9].

Concerning the last point, the phenotype(s) of CTCs has not been fully defined yet. To date, it is generally accepted that CTCs are heterogeneous, rare events of epithelial origin, among which hide out viable metastatic precursor cells capable of initiating a clonal metastatic lesion. It is currently accepted that CTCs are EpCAM+, cytokeratin (CK)+, and CD45−; however, the use of EpCAM for enriching CTCs is a matter of debate, since EpCAM biological role is controversial during carcinogenesis [10]. In fact, EpCAM over-expression has been associated with both decreased and increased survival of patients [10], whereas the protein is highly expressed in cancer stem cells in breast, colorectal and pancreatic tumors [11]. Hopefully, the scientific community is going to define a common set of criteria describing this critical district of the malignancies. However, this strictly depends on increasing knowledge regarding the biology of CTCs. The meaning of any malignant feature of CTCs should be judged according to the degree of clinical validation of a certain phenotypical characteristic that we have been using to identify CTCs in peripheral blood [12]. Furthermore, a still open question that raises doubts about the metastatic potential of CTCs is their high grade of apoptosis [13, 14].

To address these questions, we investigated whether CTCs isolated *ex vivo* from metastatic prostate and breast cancer patients were able to grow in NOD/SCID mice, subcutaneously (s.c.) injected with an increasing amount of cells (ranging from 50 to 3000). The CTCs were isolated by EpCAM enrichment, using an FDA-cleared automated platform (CellSearch) that quantifies the tumor burden in peripheral blood and provides data with predictive and prognostic value [12]. In order to estimate the apoptotic fraction present in the injected cells, we also tested the CTCs for M30 expression, as previously reported [13].

This is the first report showing that even as few as 50 EpCAM-positive live CTCs from metastatic prostate cancer patients can survive and grow in a xenograft assay, putatively containing metastasis-initiating-cells (MIC).

## RESULTS

### Quantitative enrichment of CTCs for xenograft assay

**Figure 1:**
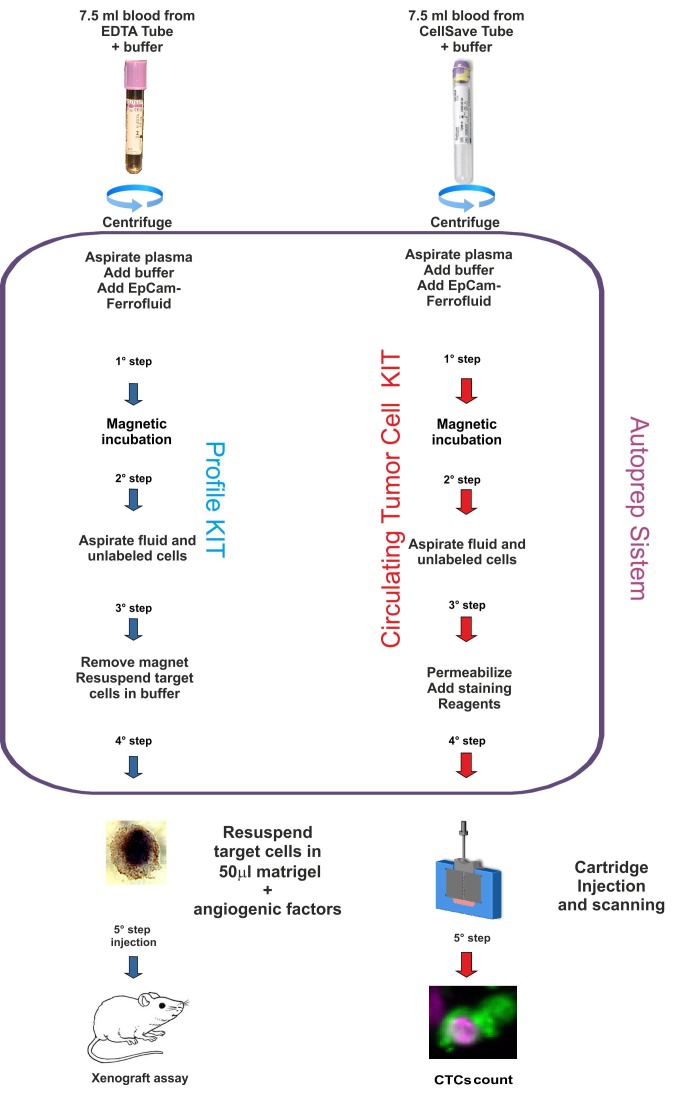
Flow chart of the experimental design The picture summarizes the main points of the procedure. Two blood draws were collected in parallel for each patient, one for the xenograft assay (EDTA tube, left part of the picture) and one for CTC count (CellSave tube, right part of the picture). The blood draws were processed online by the Autoprep, using different kits specific for EpCAM enrichment (Profile kit, light blue flux diagram) and for CTC quantification (CTC kit, red flux diagram). In the blue box the main steps of the sample automatic processing are indicated. At the end of the Autoprep run the CTCs were recovered for subcutaneously injection in mice or for fluorescence microscopy analysis (Analyzer II, CellSearch).

Among the numerous manual or semi-automated methods currently reported to enrich and/or isolate CTCs, only the CellSearch automated platform obtained the FDA approval to be used for clinical settings [3, 12], because it provides results of prognostic and predictive value with good sensibility and specificity. Therefore, we chose to enrich CTCs by CellSearch platform to develop xenograft assay. The CTCs were quantified by the platform too, starting from a second blood draw collected in parallel for every patient (Figure 1).

**Figure 2:**
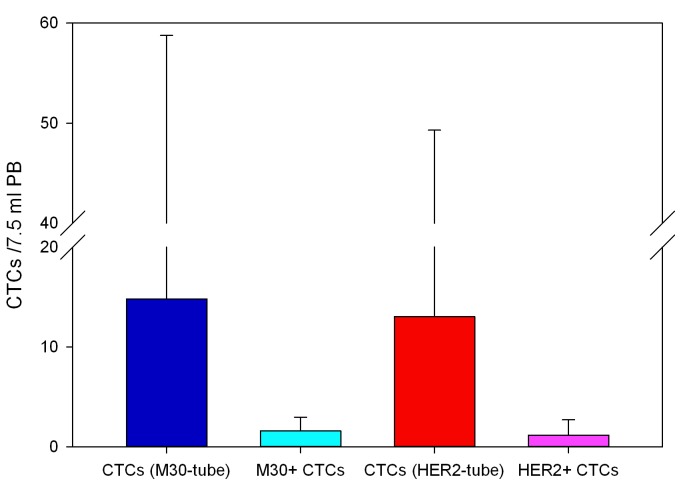
Evaluation of the between-assay variability of the CellSearch platform Twenty-two metastatic breast cancer patients were considered. The graph shows the mean total CTC number ± SD in the M30- and HER2-tube (blue and red bar, respectively) and their M30+ and HER2+ CTC fraction (light blue and pink bar, respectively). The total CTC number did not significantly differ in the two test tubes (Wilcoxon signed rank test, p= 0.706).

Since CTC enriching and counting was performed from two parallel blood draws, the between-assay variability was firstly evaluated using clinical in-house data collected from 22 Metastatic Breast Cancer (MBC) patients, tested for M30 and HER2 expression in CTCs. The mean total CTC number was 14,8 ± 43,9 cells (median 2,5 cells) in the M30-tube and 13 ± 36,2 cells (median 2 cells) in the HER2-tube, respectively (Figure 2); consistently with previous reports [15], the total CTC count in the two tubes did not significantly differ (Wilcoxon signed rank test, p= 0.706), indicating high reproducibility of the CTC assay.

### Patients' characteristics

The Table 1 (patients' data) summarizes the clinical-pathological characteristics and the baseline CTC value for the 7 enrolled patients.

**Table 1:** Clinical and pathological characteristics of CTC donor cancer patients and CTC detection in xenotransplants

Patients' data												Xenograft's data		
#	age	sex	histology	T	N	M	Gleason Score	site of metastasis	CTC/huPB (%M30+)^a^	OS^b^ days	disease status	CTC/muPB^c^	DTC/muBM^d^	IHC^e^
1	60	M	prostate adenocarcinoma	-	-	-	ND	bone	66 (2%)	297	AWD	8	1	pos
2	78	M	poorly differentiated prostate adenocarcinoma, with neural invasion, G3/G4	-	-	-	7 (3+4)	bone	264 (2%)	105	DOD	2	2	pos
3	39	F	Metastatic Breast Cancer	-	-	-	ND		402 (1%)	235	AWD	7	neg	pos
4		M	prostate adenocarcinoma, with neural invasion	2a	0	x	9 (4+5)	bone, LN	51 (6%)	270	DOD	8	3	pos
5	72	M	prostate adenocarcinoma, with neural and vascular invasion, G2	2	0	x	7 (3+4)	bone	2866 (0,2%)	404	AWD	8	1	pos
6	68	M	prostate adenocarcinoma, G3	-	-	-	8	bone	253 (3%)	384	AWD	10	neg	neg
7	see # 2	see # 2	see # 2	-	-	-			224 (0%)			42	2	neg
8	56	F	Metastatic Breast Cancer	2	2a	0	ND	bone, liver, LN, lung	207 (2%)	166	DOD	6	2	pos

a Two blood draws were collected from each patient at baseline: the first was used to determine the CTC count and the percentage of apoptotic (M30+) CTCs, the second for the xenograft assay. The numbers indicate CTC no./7.5 ml as determined by CellSearch in human peripheral blood (huPB).

b Overall Survival (OS): time occurred between CTC assessment and death or the most recent follow-up evaluation.

c At the time of euthanasia (median 10.3 months, range 6.5-12 months), murine peripheral blood (muPB) was collected and CTCs of the donor were determined as CTC no./0.75 ml by CellSearch. None of the age-matched control mice (n=6) was CTC-positive.

d Murine bone marrow (muBM) was also collected to detect the Disseminated Tumor Cells (DTCs) of donor origin by Cellsearch analysis (DTC no./0.75 ml). In the control group (n=6) the DTC mean was 0.4 ± 0.9 (median 0.0, range 0-2 DTCs).

e Results of immunohistochemistry (IHC) for human Cytokeratin in mice spleens.

After baseline assessment, the disease status of the patients was evaluated depending on the type and schedule of treatment. The median follow-up was 270 days (range 105-404 days). We observed 4 cases of alive with disease (AWD), with median Overall Survival (OS) of 340.5 days (range 235-404 days), and 3 cases of dead-of-disease (DOD), with median time-to-progression (TTP) of 166 days (range 105-270 days) (Table 1, disease status; in supplementary file). Due to the fact that all cases showed an advanced disease with a baseline CTC number higher than the cut-off value (5 cells/7.5 ml PB) for poor prognosis [3, 4], as expected, no association was found between the baseline CTC value and the patient outcome. Consistently with previous reports [13, 14], the fraction of apoptotic (M30-positive) CTCs was low (range 0-6 %, Table 1 in supplementary file) at the diagnosis of metastatic disease, before starting the therapy. Furthermore, we not found apoptotic CTCs in the sample #7, obtained at the first follow-up visit after one-month treatment; the data is consistent with the lack of any treatment response [13] and coherent with the persistent CTC number higher than 5 cells despite the treatment [3] [16].

### Human CTCs are detectable in xenografts

Eight CTC xenografts were derived from 7 consecutive patients. After a median follow-up of 10.3 months (range 6.5-12 months) none of the CTC-injected mice developed clinical evidence of tumor neither at the injection, nor at secondary sites. To assess the presence of microscopic disease in xenografts, murine peripheral blood (muPB) and bone marrow (muBM) were collected, and the presence of CTCs from donors and of Disseminated Tumor Cells (DTCs) was investigated by CellSearch, adapting the standard procedure to small blood draw.

**Figure 3:**
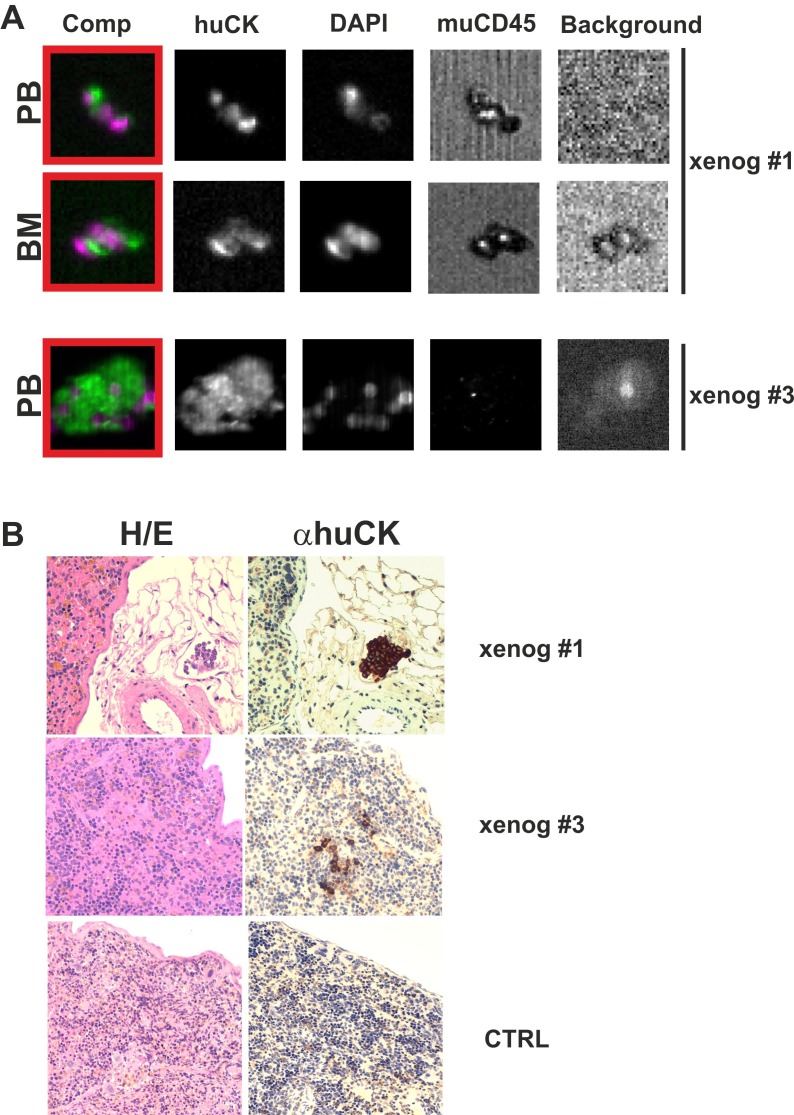
**A) Human CTCs in peripheral blood (PB) and DTCs in bone marrow (BM) of xeno-transplanted mice.** The gallery shows the same cell stained for the combination (Comp) of huCK PE (green) and DAPI (violet); huCK PE; DAPI; muCD45 APC and background control. Red squares indicate cells classified as CTCs/DTCs. **Line 1 and 2.** CellSearch analysis of some rare human CTCs in murine PB and DTCs in BM samples of a xenograft assay from prostate cancer (xenog #1). **Line 3.** CellSearch analysis of CTCs in murine PB sample of a xenograft assay from metastatic breast cancer (MBC) (xenog #3) , (Analyzer II, CellSearch). Single CTCs, exhibiting regular CK staining were detected in the PB samples of xenografts #1 and #3, whereas a small neoplastic embolus (with 3 clearly distinguished DAPI+ nuclei) was detected in the BM of xenograft #1. **B) Neoplastic cells within the spleen of xeno-transplanted mice.** The picture shows haematoxylin and eosin (H&E) staining (left panel) and pan-cytokeratin immunostaining (right panel) of murine spleens from xenografts #1 and #3 and of a mice control (original magnification, 20X). **xenog #1:** small neoplastic emboli found in perisplenic small vessels; **xenog #3:** pan-cytokeratin immunostaining disclosed small groups of anti-human cytokeratin positive cells (undetectable at the H&E staining), into the spleen red pulp; **ctrl:** the spleen from a control mouse does not display any morphological (left picture) or immunohystochemical (right picture) evidence of anti-human cytokeratin positive cells. One out of 6 representative samples is shown.

Figure 3-A illustrates an example of human CTCs and DTCs recovered from muPB and muBM of two representative xenografts, as shown by the Analyzer II device (CellSearch). As indicated in Table 1 (xenograft's data; in supplementary file), human CTCs were found in 8 out of 8 muPB samples; the mean CTC number was 11.4 ± 12.6 (median 8, range 2-42 CTCs/ 0.75 ml muPB). Moreover, human DTCs were identified in 6 out of 8 muBM samples; the mean DTC number was 1.8 ± 0.8 (median 2, range 1-3 DTCs/ 0.75 ml muBM).

Age-matched control mice (n=6) were processed in parallel and revealed no presence of human CTCs by CellSearch. Moreover, in the same control group the DTC mean was 0.4 ± 0.9 (median 0.0, range 0-2 DTCs), consistently with previous specificity reports in human samples [17].

### IHC analysis of xenografts tissues

Visual inspection did not show signs of tumor evidence in any organ or at the injection site. However, mice heart, lung, liver and spleen were paraffin embedded for immunohystochemical (IHC) analysis.

The IHC analysis showed no evidence of human CTCs in murine heart, lung, and liver. Conversely, the IHC of spleen samples for human Cytokeratin (huCK) disclosed 6 huCK-positive mice. Human CTCs were either spread as single cells into the spleen parenchyma or aggregated in small tumor emboli clearly detectable in small vessels (Figure 3-B).

Age-matched control mice (n=6) were processed in parallel and revealed no presence of human CTCs by IHC analyses.

## DISCUSSION

To our knowledge, this is the first report showing that EpCAM-positive CTCs isolated from prostate cancer patients are able to initiate metastasis in a xenograft assay [18]. This study is consistent with recent reports on xenografts derived from breast cancer [19] and hepatocellular carcinoma (HCC) CTCs [20], providing the proof of principle about the metastatic potential of EpCAM-positive CTCs. Moreover, our findings support the previous observation that, in prostate tissue, EpCAM expression significantly increases from normal tissue via prostatic intraepithelial neoplasia to adenocarcinoma [21]. Furthermore, we previously reported that EpCAM-positive live CTCs are associated with an active disease in metastatic breast cancer [13] and with progression at distant sites in metastatic renal cancer [22].

In comparison with previously reported procedures [19, 20], our xenograft assay shows some key differences that deserve to be discussed in deep.

The first item concerns the frequency of successful engraftments: for CTCs ranging from 50 to 1000, 0% (0 out of 17 mice) of positive engraftments were reported by Baccelli et al., in breast cancer patients; whereas, when more than 1000 CTCs were injected, 66.7% (6 out of 9 mice) of positive engraftments were reported [19]. On the other hand, 50% of success was reported by Sun et al., with 300 CTCs from HCC patients [20]. Our procedure is more efficient, giving 75% (6 out of 8 mice) positivity in spleen and BM, and 100% (8 out of 8 mice) positivity in PB.

Furthermore, our procedure works also when injecting a number of CTCs close to 50 cells (e.g. pts #1 and #4 in Table 1). If confirmed in larger studies, these differences may reveal a higher frequency of metastasis-initiating cells (MIC) in the CTCs collected from prostate cancer patients. Anyway, we cannot exclude that the higher efficiency of our xenograft assay might be simply due to the use of an automated platform to enrich EpCAM-positive CTCs from PB [9]. Nevertheless, the data is noteworthy, especially considering that CTC numbers >1000 cells/7.5 ml PB are relatively rare in malignancies [17], as well as CTCs >300/7.5 ml PB are rare in HCC patients [20]. This evidence would raise doubts about the relevance of mechanistic studies conducted with a large amount of CTCs, collected from a minority of advanced cancer patients, thus excluding most patients from MIC characterization analysis.

Finally, we found human CTCs in muPB, muBM and spleen samples, but we did not find signs of tumor in any inspected organ or at the injection site. These findings provide evidence that, at least in our xenograft assay, the EpCAM-positive fraction of CTCs from prostate and breast cancer patients retains the migratory capacity. This observation differs not only from xenografts derived from HCC patients, where tumors were found only at the injection site [20], but also from xenografts derived with a different procedure from breast cancer patients, where tumors arose both from the injection site and secondary sites (liver and lung) [19]. It remains to be determined, whether this difference reflects biological properties of the prostate cancer CTCs or it derives from our xenograft procedure that comprises pro-angiogenic factors in order to allow the engraftment. Nevertheless, the pro-angiogenic factors could also affect the migratory capacity of CTCs. Additional xenograft assays need to be performed in order to detect specific properties of CTCs that deserve investigation. Furthermore, it is mandatory to reach a general consensus to define successful engraftments.

In conclusion, the direct demonstration of the metastatic potential of CTCs is a critical goal for cancer research, especially in view to identify (and fight) the more invasive CTCs, that are necessary to the metastatic process [9]. The identification of a proper *in vivo* model allowing their growth represents the first step for a deeper understanding of this peculiar property.

## METHODS

### Study design

From February to August 2012, 7 metastatic cancer patients (breast cancers = 2 cases; prostate cancers = 5 cases) were consecutively enrolled for the development of a xenograft assay from human CTCs. Sequential enrollment was based on the availability of both a baseline CTC count at metastatic disease diagnosis (at least 50 CTCs/7.5 ml PB), and an informed consent for an additional blood draw to perform the xenograft assay. The disease status was evaluated at baseline, and at the end of the *in vivo* study, depending on the type and schedule of treatment (Table 1).

In one case (prostate cancer pt., #7, Table 1), a second CTC preparation for the xenograft assay was obtained at the first follow-up visit after one-month treatment.

### CTC count

The enumeration of patient's CTCs in whole blood was performed by the CellSearch^™^ System (Janssen Diagnostics) according to the manufacturer's instructions [3, 23]. An event was classified as a CTC, when its morphological features were consistent with those of a tumor cell, and it exhibited the phenotype EpCAM+, CK+, DAPI+ and CD45-. Apoptotic CTCs were also detected integrating the CTC assay with an anti-M30 monoclonal antibody (mAb). M30 is a neoepitope disclosed by caspase cleavage of cytokeratin 18 (CK18) in early apoptosis [13]. Quantitative results were expressed as the total number of CTCs and of M30-positive CTCs per 7.5 ml of blood.

The between-assay variability was tested from 22 Metastatic Breast Cancer (MBC) patients, by analyzing two different blood draw for each patient, and comparing the total CTC count in a parallel M30 and HER2 expression test.

The presence of human CTCs in murine peripheral blood (muPB), and of human disseminated tumor cells (DTCs) in murine bone marrow (muBM) was assessed by CellSearch, adapting the standard procedure to small volumes. Quantitative results were expressed per 0.75 ml of blood for both CTCs and DTCs.

For the xenograft assay, we enriched CTCs from the additional blood sample, using an anti-EpCAM mAb conjugated to ferrofluid nanoparticles (CellSearch Profile Kit, Janssen Diagnostics).

### Animals and treatments

Procedures involving animals and their care conformed to institutional guidelines that comply with national and international laws and policies (EEC Council Directive 86/609, OJ L 358, 12 December, 1987). For the xenograft assay, 8-week-old non obese diabetic/severe combined immunodeficient mice (NOD/SCID) (Charles River) were subcutaneously injected with EpCAM-enriched CTCs (CellSearch), embedded with pro-angiogenic factors (recombinant human VEGF or bFGF, 100 ng/ml; PeproTech) [24], and mixed at 4°C with liquid Matrigel (Becton-Dickinson). Mice health conditions were monitored twice weekly. The experiment was stopped in February 2013, after a median follow-up of 10.3 months (range 6.5-12 months). Heart, lung, liver and spleen were collected for IHC study; murine PB and BM were also collected for CellSearch analysis. Murine BM was collected from tibia and femurs by needle insertion into one bone end, and flush out with RPMI medium, as previously reported [25].

### Immunohistochemistry (IHC)

Fifty-six formalin-fixed, paraffin-embedded tissue samples (hearth, lung, liver and spleen) were prepared from 8 xeno-transplanted and 6 age-matched control mice. The expression of human cytokeratin was assessed by immunostaining against human pan-cytokeratin antigen (1:50 dilution, mAb clone MNF116, DAKO). The immunostaining was automatically performed according to the manufacturer's instructions (Ventana Benchmark XT system) [26]. Appropriate positive and negative controls were run concurrently.

### Statistical analysis

Data collected were analyzed by the StatGraphics software (Version 2.6), as previously reported [13]. The non-parametric Wilcoxon test was used to compare quantitative variables in the between-assay variability analysis. Spearman rank test was performed to assess the relationship between patients OS and CTC levels.

OS was measured as the time between the baseline CTC assessment (i.e. the initiation of treatment) and death. Patients who were alive at the end of the study were censored by using the time between the baseline CTC assessment and their most recent follow-up evaluation.
